# Pre-Grafting Exposure to Root-Promoting Compounds Improves Top-Grafting Performance of Citrus Trees

**DOI:** 10.3390/plants13223159

**Published:** 2024-11-10

**Authors:** Jiawei Xie, Zhihui Chen, Mohammad Naeem Lali, Huaye Xiong, Yuheng Wang, Runzheng Niu, Jingkun Zhao, Xinhua He, Yueqiang Zhang, Xiaojun Shi, Heinz Rennenberg

**Affiliations:** 1 Center of Molecular Ecophysiology (CMEP), College of Resources and Environment, Southwest University, Chongqing 400716, China; xjwswu@163.com (J.X.); xionghuaye@foxmail.com (H.X.); heinz.rennenberg@ctp.uni-freiburg.de (H.R.); 2 College of Resources and Environment, Southwest University, Chongqing 400716, China; 17723110900@163.com (Z.C.); naeem.lali97@gmail.com (M.N.L.); wyh1996@email.swu.edu.cn (Y.W.); niurunzheng@163.com (R.N.); xinhua.he@uwa.edu.au (X.H.); 3 Zhongxian Agricultural Science and Technology Extension Center, Chongqing 404300, China; 4 Department of Forestry and Natural Resources, Faculty of Agriculture, Bamyan University, Bamyan 1601, Afghanistan; 5 Interdisciplinary Research Center for Agriculture Green Development in Yangtze River Basin, College of Resources and Environment, Southwest University, Chongqing, 400716, China; 6 Chongqing Agro-Tech Extension Station, Chongqing 401121, China; zhaojk2002@163.com; 7 Beijing Changping Soil Quality National Observation and Research Station, Beijing 102200, China; 8 Chair of Tree Physiology, Institute of Forest Sciences, Albert-Ludwigs-Universität Freiburg Georges-Köhler-Allee 53/54, 79110 Freiburg, Germany

**Keywords:** citrus, top grafting, root promotion, mineral nutrient uptake, carbon and nitrogen assimilation, anti-oxidative enzyme activity

## Abstract

Top grafting is an efficient and practical technique for the renewal and rejuvenation of citrus trees in old orchards. However, root death after top grafting restricts plant growth and canopy reconstruction. Thus, applications of rooting promotion substances before citrus top grafting may increase the amount and activity of roots, thereby enhancing top-grafted plant performance. To test this assumption, four rooting promotion substances, i.e., rooting promotion powder, biochar, organic fertilizer, and potassium fulvic acid, were applied before top grafting, and the effects on biometric and physiological parameters were analyzed after top grafting. The results showed that the application of all rooting promotion substances before top grafting has a positive effect on growth and mineral nutrient acquisition, as well as on foliar C and N assimilates and the activity of anti-oxidative enzymes of top-grafted plants. Rooting promotion powder and biochar had the best effect on top-grafted tree performance in the short term. In conclusion, pre-grafting root promotion reduced root damage, enhanced nutrient acquisition, and improved the physiological performance of top-grafted plants. Therefore, this approach can play a crucial role in accelerating canopy reconstruction in old citrus orchards and in improving citrus plant development.

## 1. Introduction

Based on production and cultivation area, citrus is one of the most important fruit crops in the world, particularly in tropical and subtropical regions [1,2]. Grafting constitutes an excellent asexual propagation technique that is widely applied by the citrus industry to improve growth [3], resistance [4], and fruit yield and quality [5,6]. In recent years, top grafting has rapidly become a means to adjust the structure of citrus trees and improve market competitiveness. Top-grafting after root grafting combines three different citrus species in one to form a new hybrid with excellent characteristics, such as rapid seeding, crown formation, and high fruit yield and quality [7,8].

However, improper grafting and cultivation management often lead to the slow growth of scions, late crown establishment, and slow yield recovery. For successful grafting and appropriate scion growth, grafting time [9,10] and grafting height are essential [7,11]. In addition, management practices such as fertilization [12], bud blotting [13], and irrigation after top grafting are key factors affecting grafting success [14]. Truncated top grafting mediates an imbalance in the root-to-crown ratio and causes a large area of the root to decline [15]. However, significant root apoptosis restricts mineral nutrient uptake and constrains the growth and development of the aboveground portion of top-grafted plants, which mainly depend on the nutrition status of the root and the trunk [16,17].

In this context, substances that promote root growth, commonly used in horticulture/agriculture, may improve grafted citrus growth. Biochar, as a soil amendment, can regulate the physical structure of the soil and improve the growth environment of roots [18]. Organic fertilizer, which can decompose to produce organic acids, enhances the mineralization of organic nutrients and increases the abundance of beneficial microorganisms in the soil [19]. Potassium fulvic acid can adsorb, exchange, and activate mineral elements in soils, thereby improving soil nutrient availability. As a plant growth regulator, potassium fulvic acid can also stimulate root growth [20].

Previous studies have shown that root growth and performance of fruit trees can be effectively promoted by applying biochar [21], organic fertilizer [22], and several bio-stimulants to grafted plants [23]. Similarly, rooting powder can induce lateral root formation due to its high content of various auxins [24]. In a study with grapes, Zhou et al. [25] showed that treating rootstocks with rooting promotion powder before grafting can inhibit scion formation while promoting tissue healing and rooting, thereby enhancing grafting success. However, similar studies have not been reported for citrus trees. Therefore, we exposed the roots of citrus trees to four rooting promotion substances, namely rooting promotion powder, biochar, organic fertilizer, and potassium fulvic acid, before top grafting and analyzing the performance of the trees after top grafting. We hypothesized that these treatments (a) differently alleviate root apoptosis caused by top grafting and, thus, (b) differently accelerate nutrient uptake as well as the crown reestablishment of top-grafted citrus trees. In addition, we hypothesized that pre-grafting root promotion (c) significantly improves post-grafting tree performance by stimulating C and N assimilation and enhancing the activity of anti-oxidative enzymes in the leaves.

## 2. Results

### 2.1. Biometric Parameters

**Figure 1:**
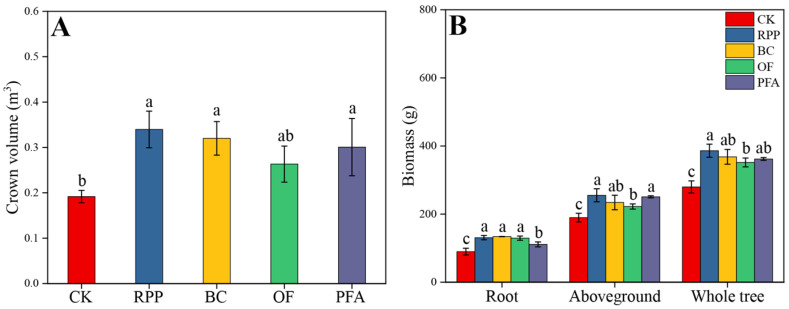
Crown volume (**A**) and biomass production (**B**) were affected by four rooting promotion substances. CK: without rooting promotion substances; RPP: rooting promotion powder; BC: biochar; OF: organic fertilizer; PFA: potassium fulvic acid. Different lowercase letters represent statistically significant differences between treatments (one-way ANOVA (Duncan), *p* < 0.05, *n* = 3).

The growth of top-grafted citrus plants was improved by rooting promotion treatments (Figure S1). Except for the organic fertilizer treatment, the other three rooting promotion substances applied before top grafting increased the crown volume of citrus plants after top grafting (Figure 1A). The largest crown volume of 0.34 m^3^ was observed in the rooting promotion powder treatment, with a significant increase of 79.0%. All rooting promotion treatments significantly increased root biomass, aboveground biomass, and whole tree biomass of citrus (Figure 1B). Biochar treatment showed the highest root biomass (134 g plant^−1^), with a significant increase of 49.1%. The aboveground biomass and whole tree biomass were highest in the rooting promotion powder treatment, with a significant increase of 34.5% and 38.0%, respectively. These results show that plants treated with rooting promotion powder or biochar had the best growth performance.

### 2.2. Root Parameters

**Figure 2:**
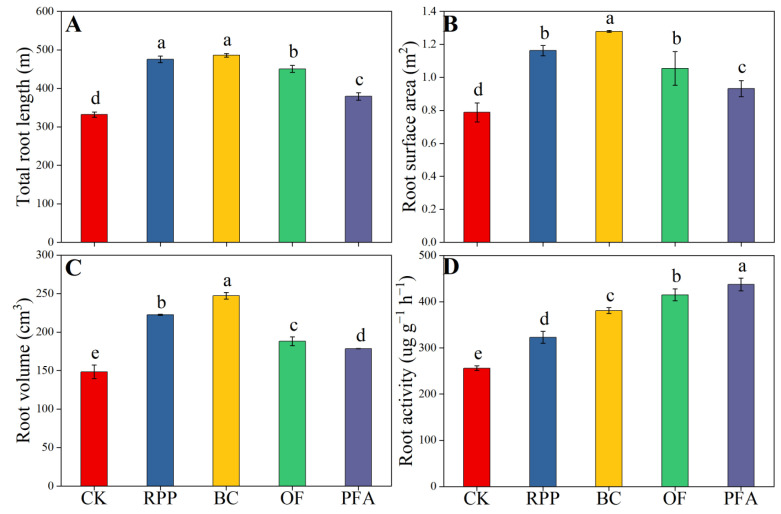
The total length (**A**), surface area (**B**), volume (**C**), and activity (**D**) of the root. CK: without rooting promotion substances; RPP: rooting promotion powder; BC: biochar; OF: organic fertilizer; PFA: potassium fulvic acid. Different lowercase letters represent statistically significant differences between treatments (one-way ANOVA (Duncan), *p* < 0.05, *n* = 3).

The four rooting promotion substances applied before top grafting significantly promoted total root length, root surface area, root volume, and root activity after top grafting (Figure 2), with the biochar treatment showing the strongest effect. Compared to the control treatment, root growth, total root length, root surface area, root volume, and root activity were significantly improved upon biochar treatment by 46.4%, 62.0%, 66.5%, and 48.7%, respectively. Thus, biochar was the best substance to enhance root growth.

### 2.3. Leaf Physiological Characteristics

#### 2.3.1. Soluble Sugar and Starch Contents

**Figure 3:**
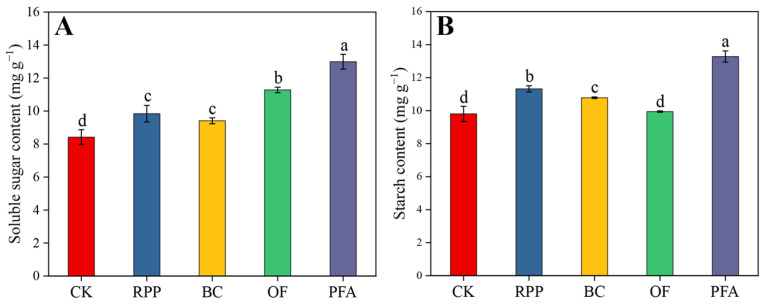
The contents of soluble sugar (**A**) and starch (**B**) in the leaves. CK: without rooting promotion substances; RPP: rooting promotion powder; BC: biochar; OF: organic fertilizer; PFA: potassium fulvic acid. Different lowercase letters represent statistically significant differences between treatments (one-way ANOVA (Duncan), *p* < 0.05, *n* = 3).

After top grafting, the leaf soluble sugar contents were significantly enhanced by the four rooting promotion treatments (Figure 3A). The leaf soluble sugar contents were highest in plants treated with potassium fulvic acid (13.0 mg g^−1^), with an increase of 54.3%. Except for the organic fertilizer application, leaf starch contents in plants subjected to the other rooting promotion treatments were significantly increased by 10.0–35.5% (Figure 3B), with the potassium fulvic acid treatment showing the highest effect. Thus, potassium fulvic acid was the best substance to increase the content of non-structural carbohydrates in leaves.

#### 2.3.2. Soluble Protein and Free Amino Acid Contents

**Figure 4:**
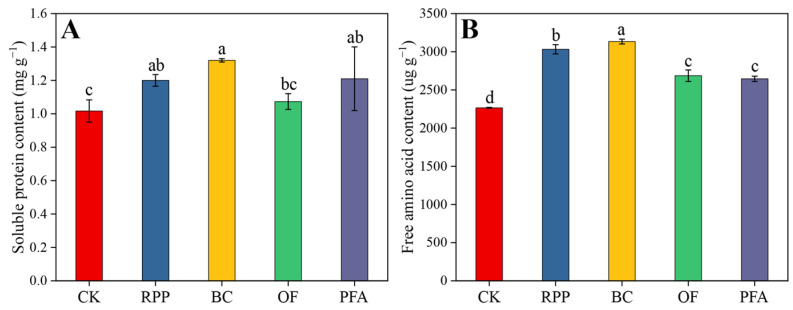
The contents of soluble protein (**A**) and free amino acid (**B**) in the leaves. CK: without rooting promotion substances; RPP: rooting promotion powder; BC: biochar; OF: organic fertilizer; PFA: potassium fulvic acid. Different lowercase letters represent statistically significant differences between treatments (one-way ANOVA (Duncan), *p* < 0.05, *n* = 3).

Except for the organic fertilizer treatment, leaf soluble protein of plants subjected to the other rooting promotion treatments was significantly higher than in controls after top grafting. Moreover, the effects of rooting promotion powder, biochar, and potassium fulvic acid did not differ significantly between each other (Figure 4A). All rooting promotion treatments significantly increased the leaf amino acid contents by 16.8–38.3%, with the biochar treatment showing the highest increase (Figure 4B). Thus, biochar was the best substance to increase the content of soluble protein and free amino acids in leaves.

#### 2.3.3. Anti-Oxidative Enzyme Activities

**Figure 5:**
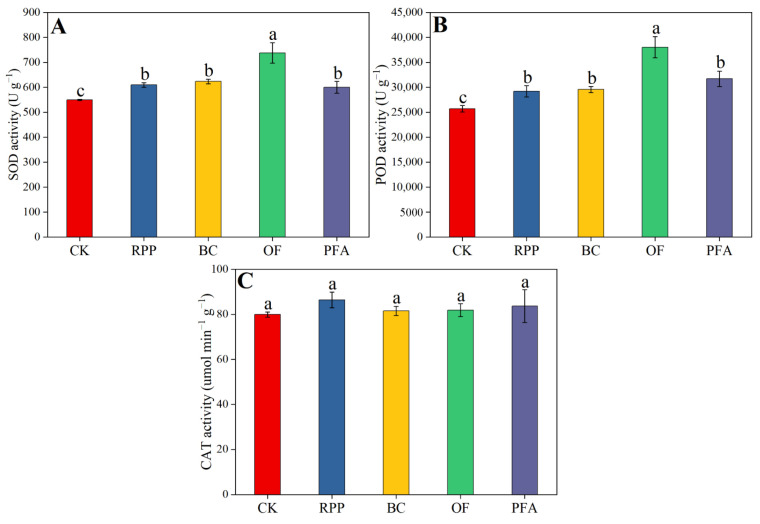
The activity of SOD (**A**), POD (**B**), and CAT (**C**) in the leaves. CK: without rooting promotion substances; RPP: rooting promotion powder; BC: biochar; OF: organic fertilizer; PFA: potassium fulvic acid. Different lowercase letters represent statistically significant differences between treatments (one-way ANOVA (Duncan), *p* < 0.05, *n* = 3).

Leaf SOD and POD enzyme activities were enhanced by all rooting promotion treatments, but this increase did not differ between rooting promotion powder, biochar, and potassium fulvic acid and was highest in the organic fertilizer treatment. Relative to the treatments with rooting promotion powder, biochar, and potassium fulvic acid, organic fertilizer significantly improved leaf SOD and POD enzyme activities by 18.4–23.0% (Figure 5A) and 20.0–30.3% (Figure 5B), respectively. The leaf CAT enzyme activity was not affected by the pre-grafting treatments (Figure 5C). Thus, organic fertilizer mediated the strongest increase in the anti-oxidant enzyme activity of citrus leaves.

### 2.4. Plant Nutrient Composition

#### 2.4.1. Plant Nutrient Concentrations

The effect of root-promoting substances on the mineral nutrient content of the top-grafted citrus tree is shown in Table 1. In roots, biochar treatment significantly enhanced P concentrations of 1.98 g kg^−1^. Biochar and potassium fulvic acid significantly increased root K concentrations by 77.2% and 68.3%, respectively. In addition, significantly enhanced root Ca concentrations of 27.2% were found in the organic fertilizer treatment. In stems, rooting promotion powder and organic fertilizer significantly increased the K concentrations by 7.62% and 9.93%, respectively, while biochar treatment significantly decreased stem K concentrations by 5.87%. In branches, the P concentrations of branches treated with organic fertilizer were significantly increased by 10.6%. Potassium fulvic acid significantly increased the branch K concentrations by 10.8%. Rooting promotion powder and biochar significantly increased the branch Ca concentrations by 1.21% and 4.59%, respectively. In leaves, total N concentrations of plants treated with rooting promotion powder significantly increased by 7.40%. Except for potassium fulvic acid, the other three rooting promotion treatments markedly improved leaf Ca concentrations by 13.0–28.8%. However, significantly enhanced Mg concentrations of 27.4% were found in leaves with the potassium fulvic acid treatment. Thus, rooting promotion powder can increase the concentration of several nutrients in citrus stems, branches, and leaves; and biochar and potassium humic acid in citrus roots, branches, and leaves. Organic fertilizer increased the concentration of several nutrients in the whole tree.

#### 2.4.2. Plant Nutrient Accumulation

Rooting promotion powder, biochar, and organic fertilizer significantly increased the root accumulation of total N by 51.3–62.9%, of P by 59.2–108%, and of Ca by 59.1–84.4% (Figure S2A,D,J). Except for organic fertilizer, there was a significant increase in root K accumulation by the other three rooting promotion treatments by 106–164% (Figure S2G). All four rooting promotion treatments, significantly improved root Mg accumulation by 38.8–59.1% (Figure S2M), as well as aboveground total N, P, K, and Ca accumulation by 24.0–44.8%, 24.5–37.2%, 20.6–45.8%, and 34.6–43.7%, respectively (Figure S2B,E,H,K). Except for organic fertilizer, aboveground Mg accumulation was significantly enhanced by 26.2–47.9% in the other three rooting promotion treatments. All rooting promotion treatments significantly improved the whole tree total N, P, K, Ca, and Mg accumulation by 33.6–49.0%, 33.7–62.6%, 31.1–60.6%, 38.7–55.6%, and 37.1–52.3%, respectively (Figure S2C,F,I,L,O). In summary, the four rooting promotion treatments increase the nutrient accumulation in roots, aboveground biomass, and the whole tree, but at different degrees.

#### 2.4.3. Plant Nutrient Distribution

**Figure 6:**
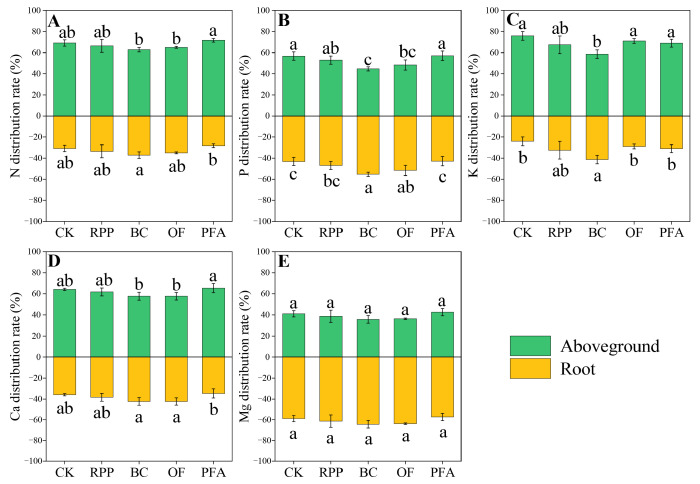
The N (**A**), P (**B**), K (**C**), Ca (**D**), and Mg (**E**) distribution of the top-grafted plant. CK: without rooting promotion substances; RPP: rooting promotion powder; BC: biochar; OF: organic fertilizer; PFA: potassium fulvic acid. Different lowercase letters represent statistically significant differences between treatments (one-way ANOVA (Duncan), *p* < 0.05, *n* = 3).

In citrus trees treated with biochar, total N distribution was increased in favor of the roots by 20.56% compared to the control (Figure 6A). Biochar and organic fertilizer significantly increased the P distribution in favor of the roots by 12.2% and 8.46%, respectively, but there was no significant effect of the other two rooting promotion treatments (Figure 6B). However, the biochar treatment significantly enhanced the K distribution in favor of the roots by 17.3% (Figure 6C). However, in citrus trees treated with biochar and organic fertilizer, higher Ca distribution was increased in favor of the roots by 10.3 to 22.2% and 10.5 to 22.3%, respectively (Figure 6D). Thus, the rooting promotion treatments enhanced the distribution of specific nutrients in favor of the roots but not in favor of aboveground biomass.

### 2.5. Cluster and Comprehensive Effects Analyses

**Figure 7:**
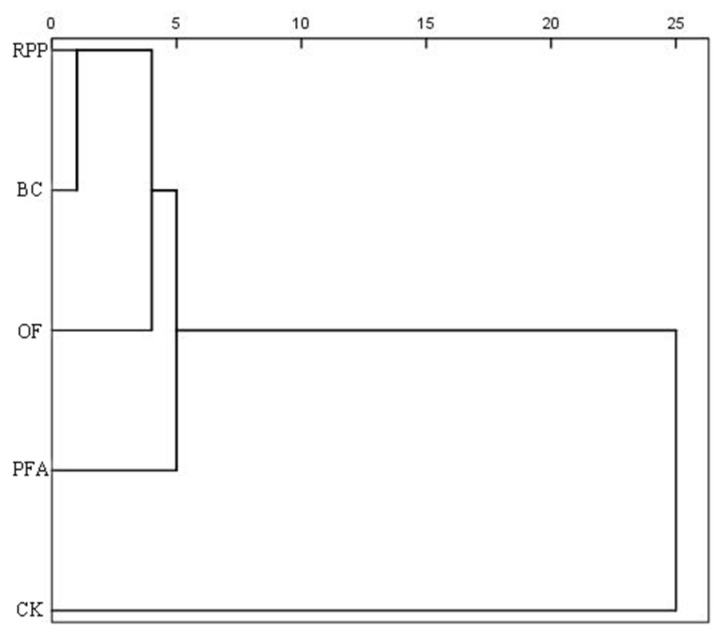
Cluster analysis of different rooting promotion treatments on the growth, physiology, and nutrient accumulation of citrus after top grafting. CK: without rooting promotion substances; RPP: rooting promotion powder; BC: biochar; OF: organic fertilizer; PFA: potassium fulvic acid.

Cluster analysis revealed that the five treatments were categorized into two main clusters (Figure 7). The first cluster comprised the four rooting promotion treatments, accounting for 80% of all treatments, while the second cluster represented the control, making up 20%. These results indicated that rooting promotion substances had significant effects on top-grafted plant growth, physiology, and nutrient accumulation.

To further assess the comprehensive effects, a coefficient of variation analysis was performed on the five treatments, and composite scores were ranked (Table 2). The results indicated that treatments with rooting promotion powder and biochar had the most favorable outcomes among the four rooting promotion treatments, with composite scores of 0.65 and 0.64, respectively.

### 2.6. Correlation Analysis and Partial Least Squares Path Model Analysis

**Figure 8:**
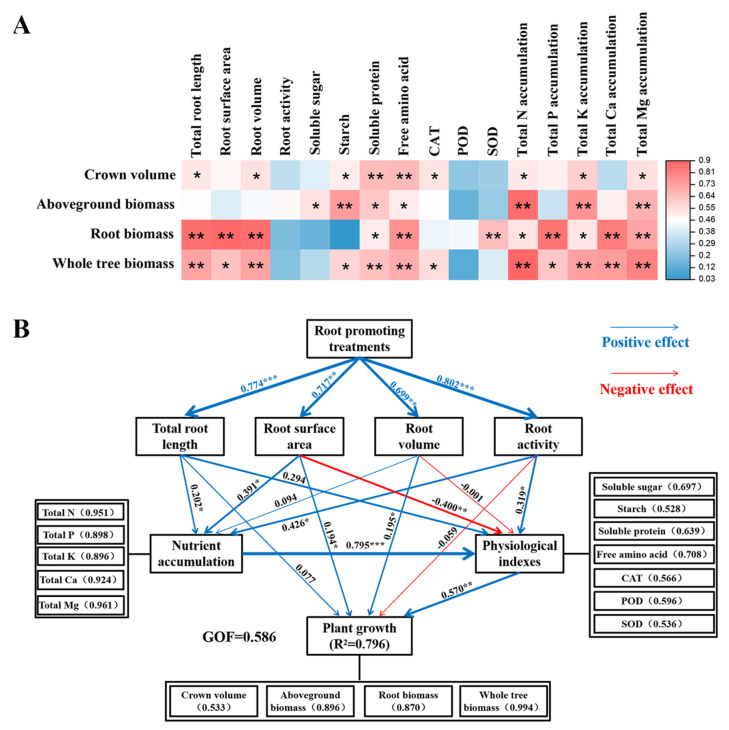
Correlation analysis (**A**) and partial least squares path model (PLS-PM) analysis (**B**) of citrus growth under different treatments before top grafting. Colors and asterisks represent the magnitude of correlation (* indicates significance at *p* < 0.05, ** indicates significance at *p* < 0.01, *** indicates significance at *p* < 0.001). Numbers within parentheses correspond to specific loading values, and GOF represents the overall fit of the model.

Correlation analysis revealed that canopy size and biomass production were significantly positively correlated with the morphological structure of the root system and most leaf physiological indicators, such as starch, soluble protein, and free amino acids. Moreover, there was a significantly positive correlation between citrus nutrient accumulation and growth indicators (Figure 8A).

To further explore the mechanism by which root-promoting substances regulate the growth of top-grafted citrus trees, after top grafting, we used the partial least squares path model (PLS-PM) to dissect the relationships among root traits, nutrient acquisition, leaf physiological indicators, and the growth of citrus plants under different rooting promotion treatments. The goodness of fit under the model was 0.586, and the PLS-PM explained 79.6% of the variation in plant growth. Rooting treatments significantly influenced root-related morphological traits, such as total root length, root surface area, root activity, and nutrient acquisition. Root activity and nutrient acquisition significantly affected leaf physiological and biochemical characteristics. Moreover, root surface area, root volume, and leaf physiological and biochemical traits directly affected plant growth. Thus, PLS-PM analyses indicated that rooting promotion treatments had a direct impact on root traits. Root development modified leaf physiological and biochemical characteristics through influencing nutrient acquisition, thereby improving plant growth (Figure 8B).

## 3. Discussion

### 3.1. Root Promotion Before Top Grafting Supports Root Growth of Top-Grafted Citrus Trees

Consistent with our hypothesis (a), the present research results indicate that pre-grafting root promotion can improve the post-grafting growth of roots. This suggests that rooting-promoting substances may effectively alleviate root apoptosis caused by top grafting through different root-promoting mechanisms. Rooting promotion powder significantly affected root morphology after top grafting (Figure 2). Previous studies showed that rooting promotion powder contains various growth-promoting hormones such as IAA, NAA, and IBA [26]. Apparently, the auxins present in rooting promotion powder affect root morphology by inducing lateral root production and adventitious root formation [24,27,28,29], thereby promoting root growth and significantly increasing root length, surface area, and volume [30,31,32]. However, the root activity in the rooting promotion powder treatment was significantly lower than in the other rooting promotion treatments of this study (Figure 2D). This might be due to the time-limited efficacy of the rooting promotion powder, with the root-promoting effect diminishing over time. In the present study, the most noticeable improvement in root traits was observed by the application of biochar. This effect can be attributed to the porous nature of biochar, which reduces substrate bulk density, increases porosity, and enhances substrate permeability [18], thereby improving the growth environment of roots and boosting root growth and vitality. Organic fertilizer treatment exhibited weaker effects on root traits compared to rooting promotion powder and biochar treatments (Figure 2A–C). This could be due to the fact that organic fertilizer constitutes a slow-release fertilizer [19], and its maximum root-promoting effect might only be realized in the long run. Still, organic fertilizer significantly increased root activity (Figure 2D), which is consistent with previous observations in sugar beet [33]. This result can be attributed to the presence of active substances, such as amino acids, humic acids, and beneficial microorganisms, that stimulate root physiological characteristics. In the present study, the increase in root activity by the application of potassium fulvic acid was significantly higher than in the other treatments (Figure 2D), possibly due to the influence of this compound on plant hormone metabolism and the concomitant regulation of root growth [34].

### 3.2. Rooting Promotion Before Top Grafting Enhances Nutrient Acquisition of Top-Grafted Citrus Trees

Consistent with our hypothesis (b), pre-grafting root promotion effectively improved post-grafting nutrient acquisition and the crown reconstruction of citrus trees. PLS-PM analysis indicated a significantly positive correlation between root traits and plant nutrient acquisition, suggesting that rooting promotion treatments enhanced nutrient accumulation by improving the root development of top-grafted citrus (Figure 8B). Overall, rooting promotion substances increased the acquisition of N, P, K, Ca, and Mg (Figure S2). This may be attributed to hormone regulation [26], soil adsorption [35,36,37,38], and soil fertilization effects of the root-promoting substances. Rooting promotion powder had the most significant effects on total N accumulation (Figure S2A–C). Du et al. [39] suggested that growth hormones could positively regulate N remobilization in cucumber. The application of rooting promotion powder might elevate plant hormone levels, thereby enhancing the efficiency of N accumulation and utilization in plants. The increased K distribution in favor of the root part (Figure 6C) confirmed that biochar could serve as a valuable potassium source, providing plants with a high concentration of exchangeable K [40]. Furthermore, biochar application promoted P acquisition by the roots and significantly increased P distribution (Figure 6B) in favor of the roots and P accumulation in both aboveground biomass and roots (Figure S2). From previous studies in herbaceous plants, it was concluded that the porosity and surface characteristics of biochar provided a favorable habitat for the growth and reproduction of soil microorganisms, reducing competition among microorganisms and protecting beneficial soil microorganisms, especially mycorrhizal fungi [41,42,43]. This is of particular significance since numerous nutrients, such as P, N, and Zn, are not only directly absorbed by roots but also indirectly through mycorrhizal fungi. Nutrients absorbed and accumulated by plants through acquisition by mycorrhizal fungi can account for 30% to 70% of the total nutrient accumulation [44,45]. According to the results of grain crop studies, the decomposition of organic fertilizer produces organic acids that enhance the mineralization of organic nutrients in the soil, thereby promoting the decomposition of insoluble nutrients, increasing plant-available nutrients, and effectively improving mineral nutrition [46,47]. The application of organic fertilizer particularly increased the content of plant-available phosphorus in the soil [48], thereby enhancing P acquisition by the roots and increasing P distribution in favor of the roots (Figure 6B). The application of potassium fulvic acid increased the absorption and accumulation of nutrients, especially of K, by citrus plants in the present study (Figure S2G–I). This effect can be attributed to the interaction of potassium fulvic acid with organic matter, oxides, hydroxides, minerals, and metal ions in the soil, effectively enhancing the availability of soil nutrients and promoting plant nutrient accumulation and utilization [20,49,50].

### 3.3. Rooting Promotion Before Top Grafting Affects Leaf Physiology of Top-Grafted Citrus Trees

The application of rooting promotion powder effectively stimulated C and N assimilation in top-grafted citrus leaves (Figure 3 and Figure 4). In previous studies of *Tamarix chinensis* and tobacco, this effect was attributed to increased chlorophyll contents [51,52], thereby improving photosynthesis and positively affecting sugar production in leaves [53]. This is due to auxin’s regulation of chlorophyll synthesis and the formation of chloroplasts [54,55]. Additionally, IBA and IAA in rooting powder can regulate nitrogen metabolism and protein synthesis [56]. In the present study, biochar treatment significantly increased the level of carbohydrates and nitrogen-containing compounds, such as soluble proteins and free amino acids, in the leaves of top-grafted citrus trees (Figure 4). Also, previous studies showed that biochar can increase soil C and N content [57] and improve the availability of nutrients in the soil [58]. In addition, our study demonstrated that organic fertilizer significantly increased the soluble sugar content in leaves (Figure 3), consistent with previous results [59]. Particularly, the combined use of organic and inorganic fertilizers can increase the photosynthetic rate and sucrose phosphate synthase (SPS) activity in leaves, leading to enhanced sucrose accumulation [60]. In the present potassium fulvic acid treatment, leaf soluble sugar and starch contents were significantly higher than in other treatments (Figure 3). A previous study in maize indicated that potassium fulvic acid directly affects CO_2_ assimilation and promotes carbohydrate accumulation by increasing phosphoenolpyruvate carboxylase (PEPC) and pyruvate orthophosphate dikinase (PPDK) activity at the transcriptional and translational levels [61].

As observed in the present study (Figure 5), a moderate rooting promotion powder treatment in *Tamarix chinensis* was previously shown to increase the activity of superoxide dismutase and peroxidase in leaves [51]. This effect can be interpreted either as oxidative stress by the rooting promotion powder treatment or as the induction of improved stress resistance. However, due to the time-limited nature of a single application of rooting promotion powder, its effect was significantly lower than in the organic fertilizer treatment. Also, biochar increased the activity of leaf superoxide dismutase and peroxidase in the present study. Zhang et al. [62] reported that the addition of biochar to the soil helps to alleviate the toxicity of organic and inorganic pollutants and enhances the anti-oxidant activity of maize. However, the organic fertilizer treatment exhibited the highest activity of superoxide dismutase and peroxidase in the leaves of top-grafted citrus trees (Figure 5), suggesting that organic fertilizer either posed particularly high oxidative stress to the plants or provided plants with particularly high stress resistance. The latter can be explained by beneficial microorganisms contained in organic fertilizers that enhance plant resistance to biotic or abiotic stress [63,64]. In the present study, anti-oxidative enzyme activities were significantly increased in the potassium fulvic acid treatment as compared to the control (Figure 5). Several studies previously showed that the application of potassium fulvic acid is a promising approach to mitigating abiotic stress in plants [65,66].

Consistent with our hypothesis (c), pre-grafting rooting promotion effectively stimulated C and N assimilation and enhanced the activity of anti-oxidative enzymes in the leaves. PLS-PM analysis of the present results indicates a significant positive correlation between nutrient acquisition and leaf physiological indicators in top-grafted citrus trees (Figure 8B). Numerous studies suggest that the balance of essential nutrients is closely related to plant carbon [67,68] and nitrogen metabolism [69] and participates in the regulation of anti-oxidant enzyme activity [70]. Therefore, improved mineral nutrition by the pre-grafting application of rooting promotion substances may have improved C and N metabolism and strengthened the anti-oxidative capacity of top-grafted citrus trees in the present study.

### 3.4. Rooting Promotion Before Top Grafting Improves Growth of Top-Grafted Citrus Trees

The application of rooting promotion substances significantly increased both aboveground and root biomass. The application of rooting promotion powder particularly promoted crown volume and aboveground biomass (Figure 1). Also, Parađiković et al. [71] reported that the application of rooting promotion powder significantly increased the plant height, leaf number, fresh weight, and dry weight of medicinal plants. Moreover, naphthylacetic acid, a major component of rooting promotion powder, was shown to significantly promote the plant height, crown volume, and stem and leaf biomass of strawberry [72]. In the present study, the effect of the pre-grafting promotion of rooting on aboveground biomass after top grafting was most significant in the rooting promotion powder treatment (Figure 1), indicating a superior influence of growth hormones in rooting promotion powder for the regulation of the growth and development of aboveground biomass compared to the other rooting promotion treatments.

De Souza Laurentino et al. [73] determined that the optimal dose of biochar for melon seedling development is 12 t ha^−1^ by the measurement of seedling biomass and root length. In the present study, the effect of biochar treatment on root biomass was most significant among the four rooting promotion treatments (Figure 1). This can be attributed to the influence of biochar application on the growth and distribution of roots in the soil, which enhances soil nutrient availability and fertility [74] and improves nutrient accumulation efficiency. Consistent with the results of the present study (Figure 1), studies on maize and citrus indicated that the application of appropriate amounts of organic fertilizer and potassium fulvic acid can increase both aboveground and root biomass [75,76]. Correlation analysis of the present data indicated that leaf physiology and nutrient acquisition are significantly positively correlated with citrus growth (Figure 8A). PLS-PM analysis further suggested that enhanced nutrient acquisition improves plant growth by affecting leaf physiology (Figure 8B). Thus, rooting promotion substances improve plant nutrient acquisition, thereby affecting not only root but also aboveground biomass traits that regulate plant growth after top grafting.

The application of top grafting has rapidly become a means of adjusting the structure of citrus varieties and improving their market competitiveness [8]. This technology causes, however, an imbalanced root-to-crown ratio and a significant decline in root biomass [15]. More seriously, a large number of citrus orchards did not pay attention to root strengthening management before top grafting, making it difficult for roots to recover. Using biochar [21], organic fertilizer [22], and some biostimulants constitutes an effective way to improve the growth of top-grafted plants [23]. In our study, all four rooting promotion substances increased the growth and mineral nutrient acquisition, as well as the foliar C and N assimilation and activity, of anti-oxidative enzymes of top-grafted plants. However, the degree of rooting promotion depended on the properties and mechanisms of different rooting promotion substances. Therefore, based on the present results, we recommend using rooting promotion powder and biochar before top grafting as an effective strategy to maintain the root-to-crown balance and, hence, the performance of citrus trees.

## 4. Materials and Methods

### 4.1. Site Description and Experimental Setup

The experiment was conducted in Shuangbai Village (107.6694° E, 30.2847° N, 465.7 m asl), Xinli Town, Zhong County, Chongqing, southwest China, from July 2020 to October 2021. This region has a subtropical monsoon climate with a mean annual temperature of 17 °C, a mean annual sunshine of 1327 h, and a mean annual precipitation of 1279 mm. For potted plant growth, an acidic purple soil with pH 5.04, organic matter 12.7 g kg^−1^, total nitrogen 0.5 g kg^−1^, available phosphorus 50.5 mg kg^−1^, available potassium 115 mg kg^−1^, exchangeable calcium 2311 mg kg^−1^, exchangeable magnesium 402 mg kg^−1^, available copper 1.80 mg kg^−1^, available iron 49.8 mg kg^−1^, available manganese 50.9 mg kg^−1^, and available Zn 3.19 mg kg^−1^ was used.

### 4.2. Experimental Treatments

**Figure 9:**
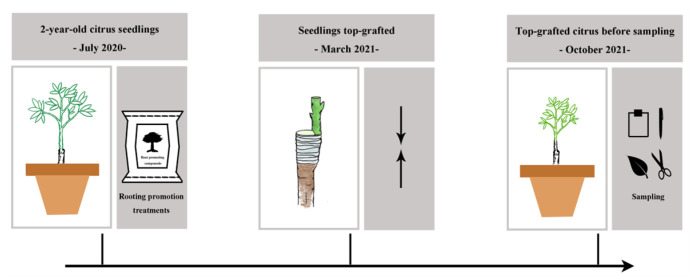
Experimental timeline. In July 2020, two-year-old citrus seedlings of *Citrus reticulate* L. grafted on *C. junos* (Sieb.) Tanaka rootstocks were planted in plastic pots. Then, plants were treated with four rooting promotion substances. In March 2021, all citrus trees were top grafted with scions of *Citrus reticulata* ‘Ai Yuan’. In October 2021, top-grafted plants were sampled.

Two-year-old citrus seedlings of *Citrus reticulate* L. grafted on *C. junos* (Sieb.) Tanaka rootstocks were planted in July 2020 in plastic pots with a top and bottom size of 38 and 30 cm diameter, respectively, and 40 cm height; 35 kg soil was used for each pot. Plants were treated with one of four rooting promotion substances: 2000 mg L^−1^ rooting promotion powder (2 g rooting promotion powder per plant; refer to Pirlak and Çinar [77] and product description), 2% biochar (0.7 kg biochar per plant; refer to Zhang et al. [78] and product description), 0.7 kg organic fertilizer per plant (refer to Zhang et al. [79] and make adjustments), and 44.6 g potassium fulvic acid (refer to product description), and without any of the above-mentioned rooting promotion substances for the control (CK). Rooting promotion powder was provided by Hebei Dewodo Biotechnology Co., Ltd. (Hengshui, Hebei, China). Biochar was provided by Zhengzhou Haosen Environmental Protection Technology Co., Ltd. (Zhengzhou, Henan, China). Organic fertilizer was provided by Beijing Goldenway Biology Tech Co., Ltd. (Beijing, China). Potassium fulvic acid was provided by Yinhai Chemical Co., Ltd. (Zhengzhou, Henan, China). Each of these treatments was replicated three times. All citrus trees were top grafted in March 2021 with scions of *Citrus reticulata* ‘Ai Yuan’ and sampled in October 2021 (Figure 9). Fertilizer applications were 150 mg N kg^−1^, 75 mg P_2_O_5_ kg^−1^, and 150 mg K_2_O kg^−1^ in 2020 and 180 mg N kg^−1^, 75 mg P_2_O_5_ kg^−1^, and 150 mg K_2_O kg^−1^ in 2021. The fertilizers used for these amendments were urea, superphosphate, and potassium sulfate.

### 4.3. Growth Parameters

The longitudinal and lateral diameters of the canopy, as well as the crown height, were separately measured with a ruler after 7 months of citrus transplanting. The canopy volume (m^3^) was calculated as follows:

V = 2/3 π r^2^ h, (1)

where V, r, and h represented the canopy volume, the canopy radius, and the canopy height, respectively.

Citrus saplings were subsequently dug out and cleaned of soil. Then, each plant was divided into the root, stem, branch, and leaf. All samples were dried at 105 °C for 30 min and then at 65 °C to constant weight for biomass determination. The aboveground biomass is the sum of stem, branch, and leaf biomass. The whole tree biomass is the sum of aboveground and root biomass.

### 4.4. Root Parameters

Newly developed white root tips were taken, and root activity was determined by the triphenyltetrazolium chloride (TTC) method [80]. Root activity primarily indicates the ability of root tissues to undergo cellular respiration and engage in active metabolic processes. Dehydrogenase in plant roots can reduce TTC to triphenylhydrazone (TTF). We recorded TTF production by the absorbance of root extracts at 485 nm to estimate dehydrogenase activity as an indicator of plant root activity. After cleaning soil from the roots, the roots were scanned with an EPSON scanner (Perfection C700, Epson Inc., Tokyo, Japan), and root morphology-related indicators (total root length, root surface area, root volume, and number of root tips) were obtained with the WinRHIZP root analysis system (Pro 2009, Rcgcnt Instrum cnt Inc., Québec, QC, Canada). Samples collected from individual plants were treated as one biological replicate, and three biological replicates were collected for each treatment.

### 4.5. Physiological Analyses

Mature leaves from spring branches were collected, snap-frozen in liquid N, and stored at −80 °C until further analyses. Total soluble sugars and starch, total soluble protein, and total free amino acids were determined by colorimetric measurements by the anthrone [81], Coomassie Brilliant Blue [82], and ninhydrin methods [83], respectively. The activities of CAT, SOD, and POD were determined with commercial test kits following the instructions of the manufacturer (Suzhou Kemin Biotechnology Co., Ltd., Suzhou, Jiangsu, China). Samples collected from individual plants were treated as one biological replicate, and three biological replicates were collected for each treatment.

### 4.6. Nutrient Content Analyses

The Kjeldahl method [84], the vanadium–molybdenum yellow colorimetric method [85], and the flame photometric method were applied to determine total N, total phosphorus, and total potassium, respectively [86], in the dried material of roots, stems, branches, and leaves. The calcium, magnesium, and other trace elements were determined using ICP-OES after the microwave digestion of tissue samples with HNO_3_-H_2_O_2_ [87].

### 4.7. Data Analyses

The following indicators were subjected to hierarchical cluster analysis: canopy volume; aboveground biomass; root biomass; whole tree biomass; total root length; root surface area, volume, and activity; leaf soluble sugar, starch, soluble protein, and amino acid contents; leaf POD, SOD, and CAT enzyme activities; and plant elemental accumulation [88]. The weight of each parameter was evaluated using the coefficient of variation method, and the comprehensive score for each treatment was calculated. To analyze the impact of different dimensions and magnitudes of indicators, the original data were subjected to min-max normalization [89].

The coefficient of variation was calculated for each indicator as shown in Equation (2):

(2) $$Cv_{j}=S_{j}/\overset{-}{x_{j}},$$

where $S_{j}$ represents the standard deviation of the jth indicator, and $\overset{-}{x_{j}}$ represents the mean of the jth indicator.

The weight of each indicator was calculated as shown in Equation (3):

(3) $$w_{j}=Cv_{j}/\sum_{i=1}^{n}Cv_{j},$$

where n is the number of evaluation indicators.

The comprehensive evaluation score was calculated as shown in Equation (4):

(4) $${Score}_{i}=\sum_{j=1}^{n}w_{j}r_{ij},$$

where $r_{ij}$ represents the normalized result of the jth indicator for the ith treatment.

One-way analysis of variance (ANOVA) was used to test the effect of each treatment on plant parameters. Differences between treatments’ means were tested with Duncan’s test (*p* < 0.05). All statistical analyses were performed using SPSS version 16.0. Graphical analysis was performed using the office 2016 and origin 2018 software. Partial least squares path modeling (PLS-PM) analysis was conducted using the “plspm” package in R (v 4.0.5) [90]. The correlation heat map between influencing factors and growth indicators was constructed using the “ggcorrplot” package [91].

## 5. Conclusions

The present results demonstrate that the application of rooting promotion substances before top grafting can enhance not only root morphology, root growth, and nutrient acquisition but can also improve the physiological and biochemical traits of aboveground biomass. The underlying mechanism by which rooting promotion treatments affect citrus growth is likely to involve the improvement in the nutrition of aboveground and root biomass, which ultimately regulates top-grafted plant growth. All four rooting promotion treatments that were studied exhibited positive effects on the traits and growth of top-grafted citrus trees, but among these treatments, rooting promotion powder and biochar application achieved the highest overall scores. With these results, the present study provides fundamental support for citrus production practices. Future studies have to elucidate whether the mixed application of several rooting promotion substances on top-grafted trees can further improve citrus growth and development and fruit production.

## Figures and Tables

**Table 1:** Effect of different treatments on the nutrient content of the top-grafted citrus. CK: without rooting promotion substances; RPP: rooting promotion powder; BC: biochar; OF: organic fertilizer; PFA: potassium fulvic acid. Data were presented as mean ± standard deviation. Different lowercase letters represent statistically significant differences between treatments (one-way ANOVA (Duncan), *p* < 0.05, *n* = 3).

Parts	Treatments	N (g kg^−1^)	P (g kg^−1^)	K (g kg^−1^)	Ca (g kg^−1^)	Mg (g kg^−1^)
Root	CK	15.45 ± 0.55 a	1.43 ± 0.25 b	5.78 ± 1.36 b	11.82 ± 0.67 b	3.15 ± 0.14 a
	RPP	17.25 ± 3.67 a	1.55 ± 0.08 b	8.25 ± 2.38 ab	12.86 ± 0.54 ab	3.43 ± 0.59 a
	BC	16.91 ± 0.29 a	1.98 ± 0.10 a	10.24 ± 0.58 a	13.85 ± 0.69 ab	3.30 ± 0.07 a
	OF	16.30 ± 0.86 a	1.73 ± 0.24 ab	6.28 ± 0.78 b	15.04 ± 2.05 a	3.24 ± 0.03 a
	PFA	16.01 ± 1.51 a	1.52 ± 0.17 b	9.73 ± 2.45 a	12.76 ± 1.81 ab	3.54 ± 0.36 a
Stem	CK	7.59 ± 0.16 a	0.62 ± 0.00 a	4.33 ± 0.10 b	10.73 ± 0.33 a	0.80 ± 0.00 a
	RPP	8.47 ± 1.07 a	0.63 ± 0.02 a	4.66 ± 0.08 a	11.26 ± 0.22 a	0.84 ± 0.02 a
	BC	7.77 ± 0.15 a	0.63 ± 0.01 a	4.09 ± 0.06 c	11.12 ± 0.32 a	0.83 ± 0.01 a
	OF	7.70 ± 0.17 a	0.64 ± 0.01 a	4.76 ± 0.22 a	12.38 ± 0.26 a	0.83 ± 0.02 a
	PFA	8.10 ± 0.44 a	0.63 ± 0.01 a	4.44 ± 0.07 b	11.52 ± 3.14 a	0.85 ± 0.04 a
Branch	CK	11.16 ± 0.64 a	1.00 ± 0.01 b	6.20 ± 0.04 b	8.28 ± 0.00 c	1.23 ± 0.02 a
	RPP	11.65 ± 0.52 a	1.03 ± 0.03 b	6.50 ± 0.03 ab	8.38 ± 0.05 b	1.30 ± 0.07 a
	BC	11.90 ± 0.14 a	1.01 ± 0.03 b	6.42 ± 0.01 ab	8.66 ± 0.05 a	1.19 ± 0.15 a
	OF	11.70 ± 0.53 a	1.10 ± 0.04 a	6.54 ± 0.34 ab	8.29 ± 0.00 c	1.31 ± 0.22 a
	PFA	12.11 ± 0.68 a	1.03 ± 0.01 b	6.87 ± 0.59 a	8.30 ± 0.02 c	1.15 ± 0.04 a
Leaf	CK	24.46 ± 0.44 b	0.93 ± 0.17 a	12.28 ± 0.15 a	10.73 ± 0.68 c	1.02 ± 0.02 b
	RPP	26.27 ± 1.20 a	0.93 ± 0.08 a	12.39 ± 0.32 a	12.12 ± 0.65 b	1.06 ± 0.03 b
	BC	24.95 ± 0.78 ab	0.99 ± 0.16 a	12.25 ± 0.34 a	12.54 ± 0.14 b	1.06 ± 0.09 b
	OF	25.74 ± 0.75 ab	0.97 ± 0.07 a	12.36 ± 0.22 a	13.82 ± 1.17 a	1.05 ± 0.04 b
	PFA	25.37 ± 0.51 ab	0.94 ± 0.01 a	12.96 ± 0.79 a	11.53 ± 0.12 bc	1.30 ± 0.01 a

**Table 2:** Comprehensive score and ranking of coefficient of variation for various rooting promotion treatments. CK: without rooting promotion substances; RPP: rooting promotion powder; BC: biochar; OF: organic fertilizer; PFA: potassium fulvic acid.

Treatment	Comprehensive Score	Ranking
CK	0.08	5
RPP	0.65	1
BC	0.64	2
OF	0.56	4
PFA	0.58	3

## Data Availability

The data that support the findings of this study are available from the corresponding author (Yueqiang Zhang) upon reasonable request.

## Supplementary Materials

The following supporting information can be downloaded at: https://www.mdpi.com/article/10.3390/plants13223159/s1, Figure S1: Top-grafted plant growth as affected by four rooting promotion substances. Figure S2: The N, P, K, Ca, and Mg accumulation of the top-grafted plant.
