# Supplementary material for: Pre-Grafting Exposure to Root-Promoting Compounds Improves Top-Grafting Performance of Citrus Trees
Source: Plants (Basel). 2024 Nov 10;13(22):3159. doi: 10.3390/plants13223159 (PMC11597673; doi:10.3390/plants13223159)
Supplement: Supplementary file 1 [file plants-13-03159-s001.zip › Supplemental Figures.pdf]

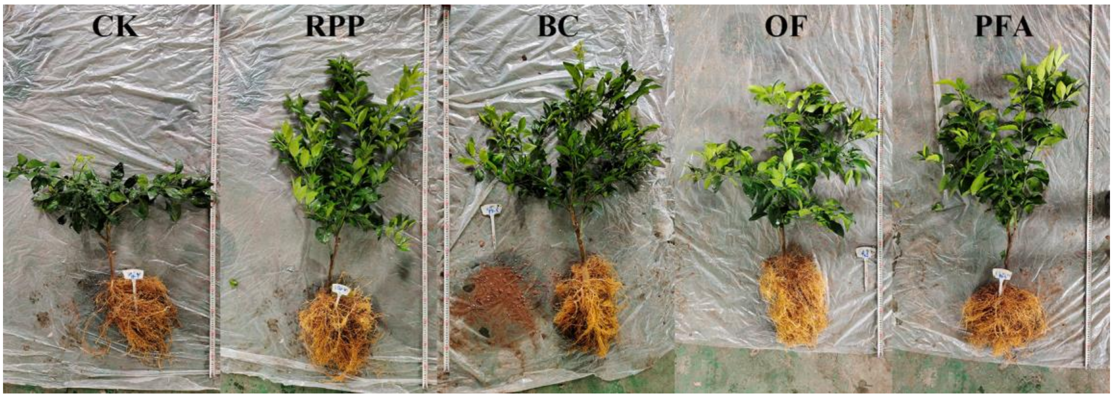

Figure S1. Top-grafted plant growth as affected by four rooting promotion substances. CK: without rooting promotion substances; RPP: rooting promotion powder; BC: biochar; OF: organic fertilizer; PFA: potassium fulvic acid.

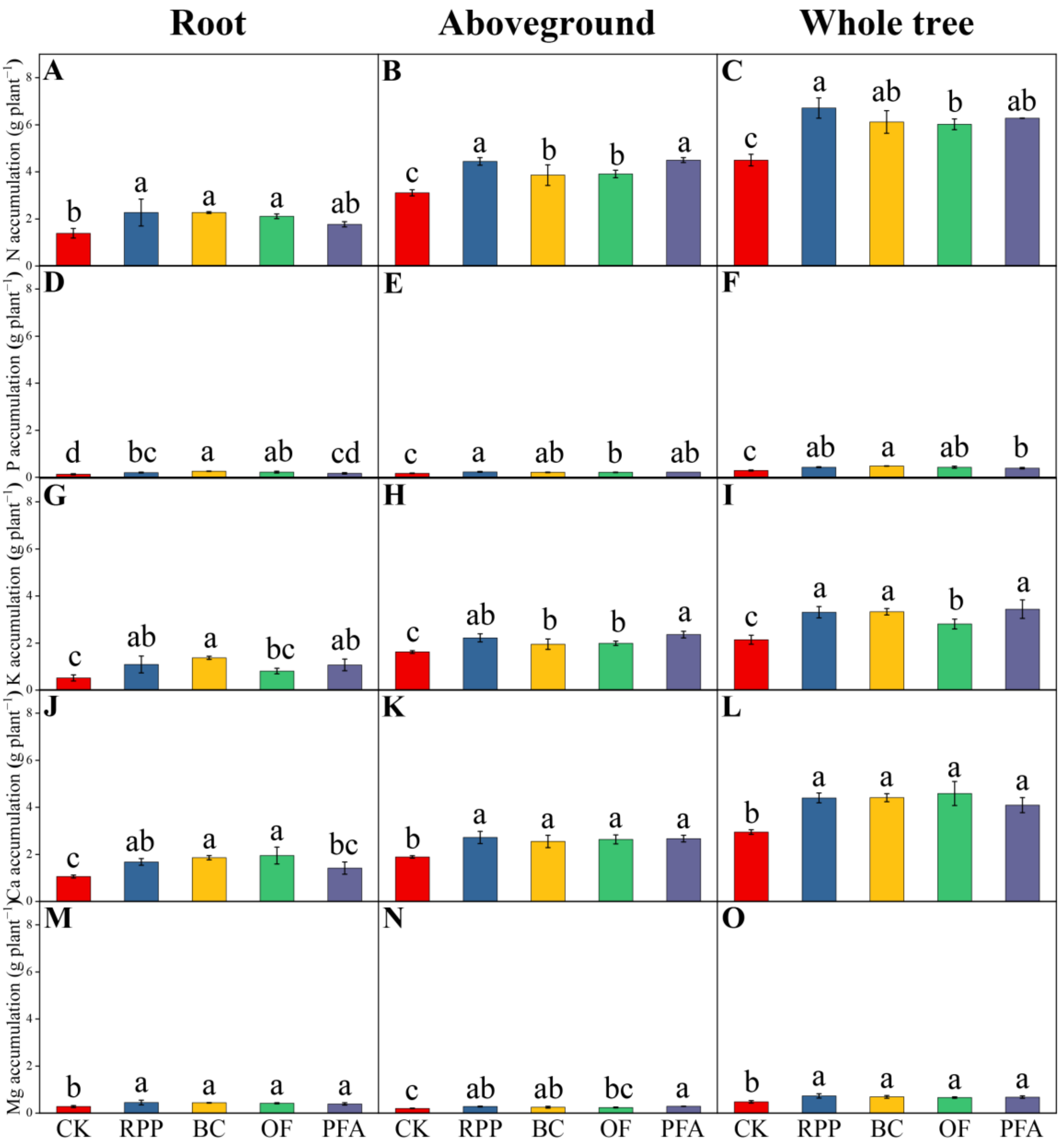

Figure S2. The N **(A-C)**, P **(D-F)**, K **(G-I)**, Ca **(J-L)** and Mg **(M-O)** accumulation of the top-grafted plant. CK: without rooting promotion substances; RPP: rooting promotion powder; BC: biochar; OF: organic fertilizer; PFA: potassium fulvic acid. Different lowercase letters represent statistically significant differences between treatments (one-way ANOVA (Duncan), $p < 0.05$, $n = 3$).
